# Optoretinography reveals rapid rod photoreceptor movement upon rhodopsin activation

**DOI:** 10.1038/s41377-025-02149-6

**Published:** 2026-01-07

**Authors:** Huakun Li, Connor E. Weiss, Vimal Prabhu Pandiyan, Davide Nanni, Teng Liu, Pei Wen Kung, Bingyao Tan, Veluchamy Amutha Barathi, Leopold Schmetterer, Ramkumar Sabesan, Tong Ling

**Affiliations:** 1 School of Chemistry, Chemical Engineering and Biotechnology, Nanyang Technological University, Singapore, Singapore; 2 Department of Ophthalmology, University of Washington School of Medicine, Seattle, WA USA; 3 Graduate Program in Neuroscience, University of Washington, Seattle, WA USA; 4 Roger and Angie Karalis Johnson Retina Center, Seattle, WA USA; 5 Department of Bioengineering, University of Washington, Seattle, WA USA; 6 Singapore Eye Research Institute, Singapore National Eye Centre, Singapore, Singapore; 7 SERI-NTU Advanced Ocular Engineering (STANCE) Program, Singapore, Singapore; 8 Ophthalmology & Visual Sciences Academic Clinical Program (EYE ACP), Duke-NUS Graduate Medical School, Singapore, Singapore; 9 Department of Ophthalmology, Yong Loo Lin School of Medicine, National University of Singapore and National University Health System, Singapore, Singapore; 10 AIER hospital group, Changsha, China; 11 Department of Clinical Pharmacology, Medical University of Vienna, Vienna, Austria; 12 Center for Medical Physics and Biomedical Engineering, Medical University of Vienna, Vienna, Austria; 13 Rothschild Foundation Hospital, Paris, France; 14 School of Electrical and Electronic Engineering, Nanyang Technological University, Singapore, Singapore

**Keywords:** Imaging and sensing, Biophotonics, Adaptive optics

## Abstract

Rod photoreceptors are essential for vision under dim light conditions and are highly vulnerable in retinal degenerative diseases. Here, we demonstrate that both human and rodent rods undergo a minute and rapid contraction of their outer segments upon photoisomerization, the first step of phototransduction. The contraction is explained as an electromechanical manifestation of the rod early receptor potential generated in the disk membranes, which is challenging to access in electrophysiology. The in vivo optical imaging of light-evoked electrical activity in rodent rods was facilitated by an ultrahigh-resolution point-scan optical coherence tomography (OCT) system, combined with an unsupervised learning approach to separate the light-evoked response of the rod outer segment tips from the retinal pigment epithelium-Bruch’s membrane complex. In humans, an adaptive optics line-scan OCT facilitated high-speed recordings in rods. The non-invasive in vivo optical imaging of rhodopsin activation extends the diagnostic capability of optoretinography, and may facilitate personalized, objective assessment of rod dysfunction and visual cycle impairment in inherited and age-related macular degeneration.

## Abstract (web summary)

Optoretinography reveals rapid deformations in human and rodent rod photoreceptors associated with rhodopsin activation, paving the way for label-free imaging of rod dysfunction and visual cycle impairment in retinal diseases.

## Graphical abstract

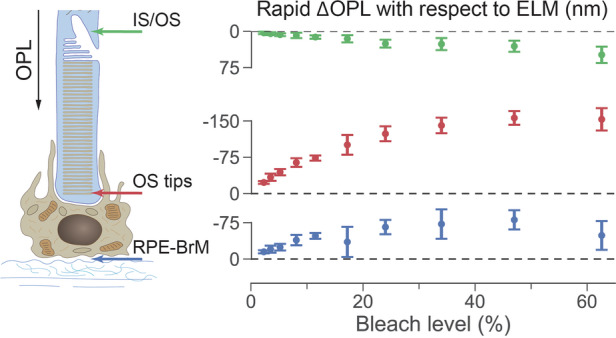

## Introduction

Vision begins with the photoisomerization of visual pigments^1^–the initial step of phototransduction in rod and cone photoreceptors. Rods, in particular, have been extensively studied for their exquisite sensitivity to detect single photons^2–4^. The early decline of rod function during normal aging^5^, age-related^6^, and inherited^7^ retinal degenerations has further emphasized the importance of this vulnerable cell population. Efforts to probe the functional capacity of rods include scotopic visual sensitivity^8^, rod-mediated dark adaptation^9^, and scotopic electroretinogram (ERG)^10^ in vivo, while patch-clamp and suction-pipette electrical recordings have been used ex vivo^4^. Scotopic perimetry and rod-mediated dark adaptation are subjective tests that evaluate patients’ visual function in low-light conditions and provide a window into the health of rods and their visual cycle^11,12^. Scotopic ERG, on the other hand, is a standardized, objective clinical test that measures rod function using corneal or conjunctival electrodes^10^. The ERG a-wave quantifies the light-evoked reduction of the dark current in the rod population. Patch-clamp and suction-pipette recordings provide detailed insights into electrical activity ex vivo, including dark current, ion channel activity, photocurrent, and graded membrane potential in rod photoreceptors^4^. The in vivo techniques are limited in their sensitivity, specificity, and cellular resolution, while the ex vivo approaches are invasive to be used in living eyes. An ideal measure of rod viability would inform us of both its structural and functional integrity with high sensitivity and resolution.

Recently, optoretinography (ORG) has emerged as a non-invasive technique to probe light-evoked photoreceptor activity, including the early steps of phototransduction, amplification, and the visual cycle^13–16^. Remarkably, phase-resolved optical coherence tomography (OCT) has enabled reliable and quantitative detection of outer segment (OS) elongation in both rod and cone photoreceptors associated with phototransduction^16–22^. Such elongation has been attributed to water influx driven by osmotic imbalance, as well as hydrolysis of the all-trans chromophore during phototransduction^16,23^. With adaptive optics (AO), these light-evoked OS responses can be localized to individual cones^18–20,24^. Interestingly, ORG has also led to the discovery that the activation of cone opsins by a strong bleaching stimulus induces a rapid (<5 ms) contraction of human cone OS preceding the later elongation^13,17,18^. The light-evoked rapid contraction of cone OS was hypothesized to result from voltage-dependent membrane tension—the electrostatic repulsions between mobile ions near the disk membrane—during the early receptor potential (ERP)^13,25^.

However, the corresponding contractile response of rod OS upon light activation has remained elusive. Previous studies have primarily focused on the light-evoked elongation of rod OS that peaks at around half a second^19,22^. Unlike cones whose disk membranes are confluent with the cell membrane and thus easily accessible for suction-pipette recordings^26^, rod disk membranes are predominantly isolated and electrically uncoupled from the plasma membrane^27–29^. Thus, conventional electrophysiology cannot readily access the ERP on the rod disk membranes, which arises from charge displacements accompanying the conformational change of visual pigments upon photoisomerization. We sought to test the feasibility of ORG as a candidate tool to access the optical manifestation of electrical activity within the rod disk membranes, which would enable non-invasive in vivo imaging of rhodopsin activation using an OCT machine. In addition, ORG signatures associated with the ERP, if detectable in rods as well, would shed new light upon the underlying biophysical mechanism. Specifically, we test the hypothesis that, like cones, rod disks exhibit an ERP that produces a rapid and minute electromechanical contraction of the OS. In Sections 2.1–2.3, we first report ORG experiments in wild-type rats using a non-AO point-scan OCT combined with unsupervised signal classification to differentiate the light-evoked movement of rod OS tips from that of the retinal pigment epithelium-Bruch’s membrane complex. In Section 2.4, we report the test of the hypothesis in humans which entailed the use of an AO line-scan OCT instrument that was able to spatially segregate the rod and cone responses in 3-dimensions.

## Results

### Rapid movements in rodent rod photoreceptors and adjacent outer retinal structures in response to a strong bleach

**Fig. 1:**
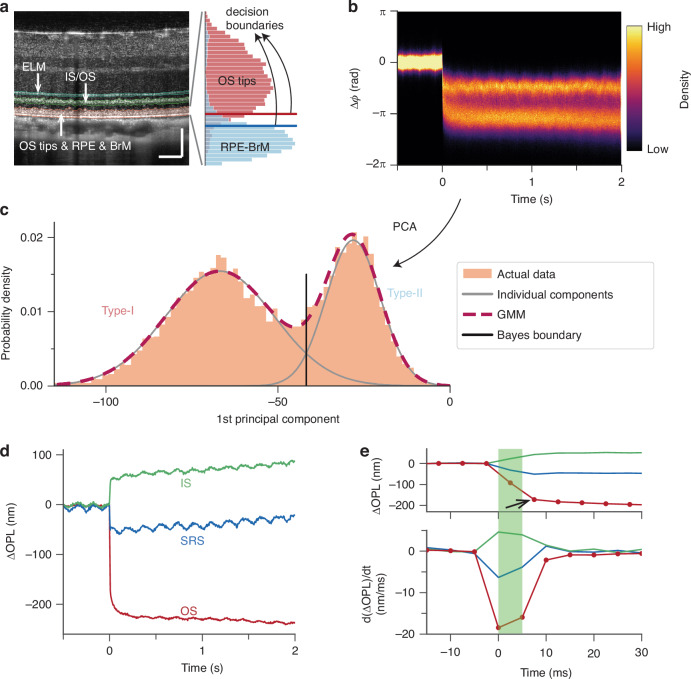
Multilayered outer retinal dynamics in response to a strong bleaching stimulus (5 ms, wavelength 505 nm, 34.0% bleach) recorded using phase-resolved OCT. **a** A representative structural image of a wild-type rat retina captured by the ultrahigh-resolution OCT. Scale bar: 100 μm. ELM: external limiting membrane; IS/OS: inner segment/outer segment junction; OS: outer segment; RPE: retinal pigment epithelium; BrM: Bruch’s membrane. Inset illustrates the isolation of OS tips and the RPE-BrM complex based on two decision boundaries (red and blue lines). **b** The distribution density of phase traces extracted from the composite layer that consists of OS tips, RPE, and BrM, by taking IS/OS as the reference. The stimulus was delivered at *t* = 0 s. **c** Each phase trace in Fig. 1b was projected onto the 1st principal component that captured 98.5% of the total variance. The distribution of the principal component score (light brown bins) was fitted with a Gaussian mixture model (GMM, dashed dark magenta line). The gray curves represent two individual components. Two types of signals were separated according to the Bayes boundary (solid black line). **d** Multilayered outer retinal dynamics in response to the visual stimulus. IS dynamics: ∆OPL between ELM and IS/OS; OS dynamics: ∆OPL between IS/OS and OS tips; SRS dynamics: ∆OPL between ELM and the RPE-BrM complex. **e** Top: An enlarged view of the initial rapid contractile response. Bottom: Time derivatives of the rapid responses. The green block represents the 5-ms visual stimulus

An OCT cross-sectional scan of the wild-type rat retina revealed several hyperreflective bands in the outer retina (colored bands in Fig. 1a), including the external limiting membrane (ELM), inner segment/outer segment junction (IS/OS), and a thick composite layer. The composite layer comprises the OS tips, retinal pigment epithelium (RPE), and Bruch’s membrane (BrM). Light-evoked dynamics at individual pixels in this composite layer can be extracted from the OCT phase signals using the IS/OS as a reference (see Materials and methods). Figure 1b shows the distribution of OCT phase signals in the composite layer in response to a 34.0% bleaching stimulus, where a rapid and pronounced decrease in the OCT phase is observed, revealing two bands with distinct amplitudes (Fig. 1b). For subsequent analysis, the temporal evolution of OCT phase was converted to a change in optical path length, ∆OPL(t), using the OCT center wavelength.

We then adopted an unsupervised learning approach, including principal component analysis (PCA) and a Gaussian mixture model (GMM), to segregate these distinct signal patterns. Specifically, all phase traces in Fig. 1b were projected onto their 1st principal component, fitted with a GMM consisting of two components (the dashed dark magenta line in Fig. 1c), and clustered into Type-I and Type-II based on posterior probability (the Bayes boundary in Fig. 1c). The depth distribution of these two signal types suggests that the Type-I signal (red bins in Fig. 1a) arose from the rod OS tips, while the Type-II signal (cyan bins in Fig. 1a) originated from the RPE-BrM complex^22^. However, at lower bleach levels, the signal patterns overlapped, and temporal features alone were less effective in separating them (Fig. S1). To establish a more reliable clustering criterion, we sought to classify signals directly based on their axial distribution (see Supplementary Section 1), as the anatomical structure along depth is expected to be consistent across measurements^30^. The red and blue lines in the inset of Fig. 1a represent optimal decision boundaries obtained by maximizing the ${F}_{\beta }$ score–a weighted harmonic mean of classification precision and recall. In this study, we set $\beta$ to 0.5 to prioritize precision over recall. Accordingly, the composite layer was separated into rod OS tips, a transition region, and the RPE-BrM complex based on normalized distance to the BrM (see Fig. S2 and the inset in Fig. 1a). In eight experiments with bleach levels ranging from 34.0% to 62.6%, we obtained an ${F}_{0.5}$ score of 0.933 (0.023) [mean (standard deviation)] for OS tips using a decision boundary of 0.421 (0.027) and an ${F}_{0.5}$ score of 0.925 (0.010) for the RPE-BrM complex using a decision boundary of 0.344 (0.026). We then used these decision boundaries to isolate OS tips and the RPE-BrM complex and calculated their respective dynamics with respect to IS/OS.

**Fig. 2:**
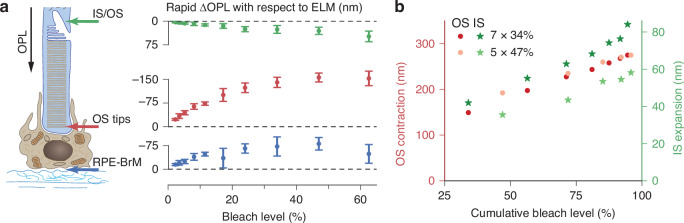
Rapid ΔOPL in the major outer retinal layers at varying bleach levels. **a** ΔOPL measured from the ELM to the IS/OS (green), OS tips (red), and the RPE-BrM complex (blue) immediately after the 5-ms flash offset. Data points and error bars represent the mean and standard deviation, respectively, at a specific bleach level. **b** The rapid movement of rod IS (green stars) and OS (red dots) in response to serial flashes (7 or 5 flashes, each yielding a 34% or 47% bleach level per flash of the remaining photopigment). The flash duration and inter-flash interval were 5 ms each. Data points (7 or 5) represent the successive OS & IS movement under the two conditions. OPL optical path length, ELM external limiting membrane, IS/OS inner segment/outer segment junction, IS inner segment, OS outer segment, RPE retinal pigment epithelium, BrM Bruch’s membrane

This multilayered adaptation of ORG allowed us to visualize the rapid light-evoked responses in the entire outer retina in a single measurement. As shown by the red curves in Fig. 1d, e, the ∆OPL of rod OS underwent a rapid decrease in response to the visual stimulus, while the IS dynamics, derived from the ∆OPL between ELM and IS/OS, displayed a rapid increase (green curves in Fig. 1d, e). The subretinal space (SRS) dynamics (spanning from the ELM to the RPE-BrM complex, depicted by the blue traces in Fig. 1d, e) exhibited a rapid ∆OPL decrease with a smaller amplitude compared to that of the OS. Unlike human cones, where the rapid OPL shrinkage of OS in response to a strong stimulus is typically short-lived (peaking at ~10 ms) and of small amplitude (<50 nm) compared to the slow OS elongation^13^, the rod OS contraction in rodents was persistent over seconds and more pronounced, reaching over 200 nm (Fig. 1d) at this bleach level. As illustrated in Fig. 2a, we further calculated the ∆OPL between the ELM and the IS/OS, OS tips, and the RPE-BrM complex immediately after the flash offset (see the time point indicated by the black arrow in Fig. 1e). We observed a contraction in OPL between (a) ELM and OS tips and (b) ELM and RPE-BrM complex during the rapid OS response. Consistent with the finding in Fig. 1d, the latter was smaller in magnitude.

In principle, the rapid OPL decrease along rod OS could result from a reduction in the physical length, a decrease in the refractive index, or a combination of both. However, as elaborated in the discussion in Fig. S3 and Supplementary Section 2, the rapid OPL changes along the OS and adjacent retinal layers cannot be explained by the refractive index change accompanying rhodopsin activation. On the other hand, they are compatible with the interpretation that such a phenomenon is primarily due to mechanical shrinkage.

We conducted multilayered ORG recordings under serial-flash stimuli to test the correspondence between the IS expansion and OS contraction. Figure 2b shows the rod IS and OS movements after each series of either 7 or 5 flashes (see Fig. S4). In both cases, each flash lasted 5 ms with an interval of 5 ms between the adjacent flashes. In total, one set of serial flashes lasted 65 ms (7 flashes + 6 intervals), and the other lasted 45 ms (5 flashes + 4 intervals), while both aimed at reaching the same cumulative bleach level (~95% bleach). Under the serial-flash stimuli, the IS expansion was correlated in its bleach dependence with the OS contraction, suggesting that these two rapid responses are initiated by the same underlying process. While both serial-flash stimuli triggered nearly identical rapid OS contractions resulting from the same cumulative bleach level, variability was observed in the amplitude of the rapid IS expansion between these two measurements. This difference could be attributed to the larger inter-individual variation observed in the IS expansion compared to the OS contraction (see Fig. S5).

#### A voltage-dependent membrane tension model explains the rapid contraction in rod OS accompanying rhodopsin activation

In previous studies on the rapid light-evoked contraction of human cone OS, a logarithmic relationship between the contraction amplitude and the stimulus strength was reported^13,25^, which is in line with the prediction of a voltage-dependent membrane tension model^25^. We investigated whether a similar model is able to explain the bleach dependence of the rapid rod OS contraction.

**Fig. 3:**
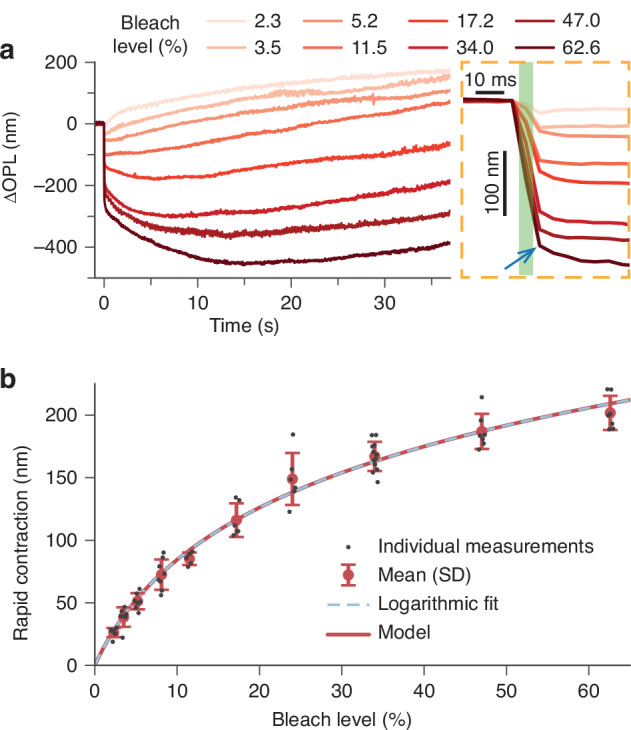
Dependence of the light-evoked rod OS dynamics on the stimulus strength. **a** Representative traces of the OS dynamics in response to flashes ranging from 2.3% to 62.6% bleach levels, with the enlarged view on the right showing an elbow point at t = 7.5 ms after the flash onset. **b** The rapid OS contraction amplitude showed a logarithmic dependence on the bleach level (dashed cyan curve). Black dots represent individual measurements. The red circles and error bars denote the mean values and corresponding standard deviation (SD) ranges at each bleach level, respectively. The red curve represents the prediction from the voltage-dependent membrane tension model

Figure 3a shows representative dynamics of the rod OS evoked by 5-ms visual stimuli at bleach levels ranging from 2.3% to 62.6%, with approximately uniform sampling on a logarithmic scale. The lower bound was selected based on the stimulus strength that can still reliably produce a rapid, albeit small (~25 nm), decrease in the OPL of rod OS, while the upper bound was determined by the maximum output power of the LED used for visual stimulation. It is important to note that the lowest bleach level used in this experiment (>2%) was still considerably higher than those used in our previous study on light-evoked rod OS elongation (<0.3% bleach level)^22^. In response to the strong bleaching stimuli, a rapid contraction was observed during the 5-ms flash stimuli (enlarged view in Fig. 3a), with the amplitude increasing with the bleach level. An elbow was consistently observed at 7.5 ms post-stimulus (the averaged timestamp of the B-scan after the flash offset, indicated by the blue arrow in Fig. 3a), where the first, rapid contractile kinetics gives way to a slower response. For bleach levels between 2.3% and 11.5%, the rapid contraction in the OS was followed by a continuous, slow elongation that persisted over the 37-second recording. Under stronger stimuli (>11.5% bleach), we observed a secondary contraction with slower kinetics than the rapid contraction in the first 7.5 ms, peaking at 5~15 s. This secondary contraction was followed by continuous elongation, similar to the signals observed under 2.3–11.5% bleach levels. For clarity in this article, we categorized these processes as rapid OS contraction (within 7.5 ms), secondary OS contraction (if any, from 7.5 ms to ~10 s), and late OS elongation.

Although the exact onset of the rapid OS contraction could not be measured due to the limitation in our imaging speed, its millisecond time scale coincides with that of rhodopsin activation, transitioning from rhodopsin to metarhodopsin II (Meta II)^1^. Previous studies have suggested that late elongation is likely associated with osmotic water influx^16,23^. For the rapid OS contraction to be similarly induced by osmolarity-driven water flow across the plasma membrane, the permeability coefficient would need to be $0.30\,\text{cm}/\text{s}$ (see Supplementary Section 3), which is much larger than the $2.6\times {10}^{-3}\text{cm}/\text{s}$ measured in in-vitro studies^31^. Thus, the observed rapid OS contraction is too fast to be induced by osmosis.

Since the rhodopsin activation is accompanied by charge displacement across disk membranes, i.e., the ERP^28,32–35^, we tested whether electromechanical coupling could explain the rapid rod OS contraction. A voltage-dependent membrane tension model was previously proposed to explain the rapid contraction in cone OS^25^ as well as cellular deformations in mammalian neurons during their millisecond-scale action potentials^36–38^. In cone and rod OS, the hyperpolarization of disk membranes during the R2 phase of the ERP would lead to an increase in the repulsive forces between mobile ions within the Debye layers^28,33,39^, causing the disk membrane to stretch. Specifically, transmembrane voltage change at the disk membranes can be calculated by,

1 $$\Delta {V}_{m}=\mathrm{bleach\; level}\times {\sigma }_{rho}\times q\,/{c}_{m}$$

where ${\sigma }_{{rho}}$ represents the area density of rhodopsin on the disk membrane, $q$ is the charge displacement per photoisomerization, and ${c}_{m}$ is the specific membrane capacitance (see Table S2). The observable membrane tension $\widetilde{\tau }$ has an initial value of ${\widetilde{\tau }}_{{\rm{rest}}}$ in its resting state and changes linearly with $\Delta {V}_{m}$, i.e., $\widetilde{\tau }={\widetilde{\tau }}_{\mathrm{rest}}+\alpha\Delta {V}_{m}$, where the coefficient $\alpha$ is -0.1 $\text{mN}/\left(\text{m}\cdot \text{V}\right)$^25,40^.

Under physiological conditions, the normalized area expansion of the disk membrane ($\Delta A/{A}_{0}$) at different membrane tensions can be calculated by^25,41^,

2 $$\frac{\Delta A}{{A}_{0}}=\frac{\widetilde{\tau }}{{K}_{A}}+\frac{{k}_{B}T}{8\pi {\kappa }_{c}}\mathrm{ln}\left(\frac{\widetilde{\tau }A/{\pi }^{2}{\kappa }_{c}+1}{\widetilde{\tau }{a}_{1}/{\pi }^{2}{\kappa }_{c}+1}\right)$$

where ${A}_{0}$ refers to the area of the disk membrane patch when the observable tension $\widetilde{\tau }$ is 0, $A$ and $\Delta A$ are tension-dependent membrane area and the corresponding area expansion, respectively, ${k}_{B}$ is the Boltzmann’s constant, $T$ is the absolute temperature (310 K). ${K}_{A}$ is the area expansion modulus, ${\kappa }_{c}$ is the bending modulus, and ${a}_{1}$ is the smallest bending feature size. In disk membranes densely packed with rhodopsin molecules, ${a}_{1}$ can be approximated to the characteristic size of the rhodopsin nanodomains (~25 nm in diameter)^42^.

Assuming volume conservation of individual disks within the millisecond-scale dynamics, the lateral expansion of individual disks would result in their axial contraction. The contraction of the rod OS during the ERP was thus modeled as the cumulative contraction of individual disks, as detailed in Supplementary Section 4. By fitting the parameters listed in Table 1, the modeled rapid contraction amplitudes matched the experimental observations across varying bleach levels (see the red curve in Fig. 3b). Furthermore, both fitted parameters (${\widetilde{\tau }}_{{\rm{rest}}}$ and ${\kappa }_{c}$) fall into the typical ranges from literature. Overall, the voltage-dependent membrane tension model can explain the observed rapid rod OS contraction as being caused by the ERP in the disk membranes.

**Table 1:** Fitting parameters for the voltage-dependent membrane tension model

Parameter	Fitted value	Typical value^25^	Unit
Initial tension, ${\widetilde{\tau }}_{\text{rest}}$	0.13 (0.05)	0.1 – 1	μN/m
Bending modulus, ${\kappa }_{c}$	$0.38\,(0.02)\times {10}^{-19}$	0.5 – 2 $\times {10}^{-19}$	$\text{N}\cdot \text{m}$

The values in the parentheses represent the standard deviations of the fitted parameters

### Rod OS responses elicited by multi-flash stimulation

**Fig. 4:**
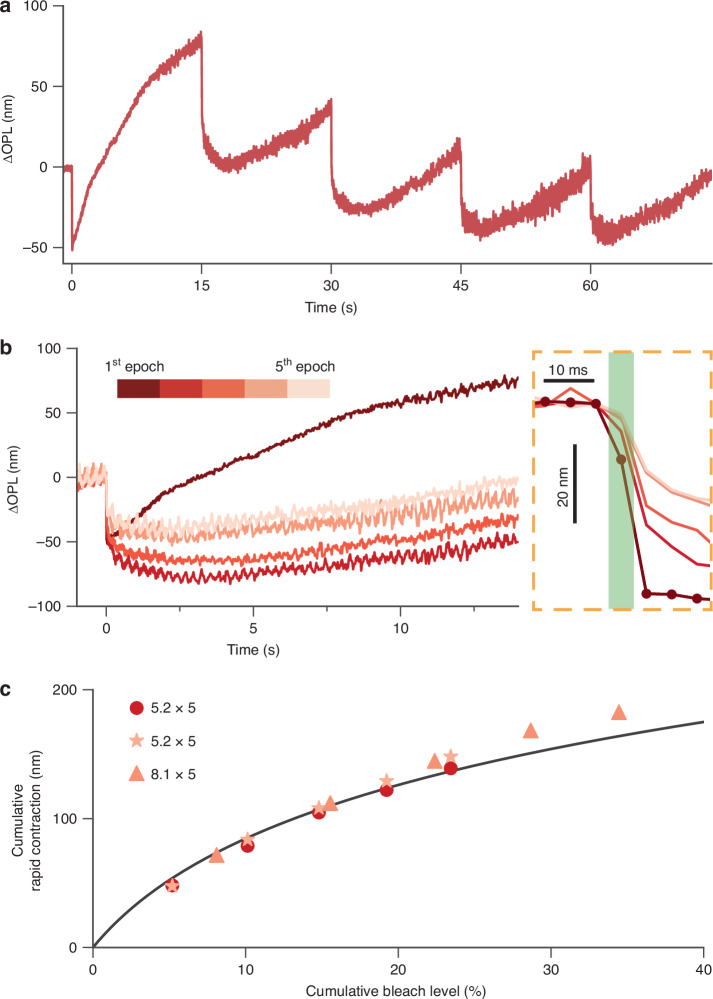
Rod OS responses evoked by 5 flashes with an inter-flash interval of 15 s. **a** Prolonged ORG recording of the rod OS responses to five flashes over 75 s, with each flash bleaching 5.2% of the remaining rhodopsins. **b** The rod OS responses extracted from each epoch were aligned to the flash onsets, with an enlarged view of the rapid OS responses shown in the dashed rectangle. **c** The cumulative rapid contraction, calculated as the sum of the rapid OS response amplitudes elicited by all flashes, was plotted against the cumulated bleach level, which represents the total percentage of rhodopsins bleached by the consecutive flashes. Dots correspond to the measurement in Fig. 4a, b, and star markers were extracted from the measurement conducted in another animal following the same protocol. Triangle markers correspond to the measurement in Fig. S6. The black curve represents the same modeled curve in Fig. 3b

Based on the hypothesis linking the rod OS contraction to the ERP within the disk membranes, it is predicted that a single flash or a set of serial flashes will both elicit the same amplitude of contraction, provided they confer the same effective rhodopsin bleach. Further, under both stimulation conditions – single or serial flashes – the saturation of the contraction amplitude should be explained by the same voltage-dependent membrane tension model. We tested this prediction by undertaking ORG with multiple flashes of equivalent strength that were separated by a longer time interval compared to Fig. 2b. Figure 4a illustrates the rod OS responses to 5 flashes with an inter-flash interval of 15 s, where each flash bleached 5.2% of the remaining rhodopsins. To better compare the rod OS responses evoked by consecutive flashes, we aligned the signals extracted from each epoch to their flash onsets (see Fig. 4b). Notably, we observed that successive flashes significantly slowed the rate of late OS elongation. The slowdown is particularly evident after the 1st flash, where only a small fraction of rhodopsin (5.2%) was bleached. Figure S6 shows another similar example with each flash bleaching 8.1% rhodopsins. This observation suggests that the late OS elongation, purported to be linked to the amplification stages of the phototransduction cascade^16,23^, is heavily suppressed by the first flash. This phenomenon resembled previous observations using electrical recordings, where a second flash delivered 1 min after the first flash could isolate the ERP from the late receptor potential^33,35^. It follows that the enzymes and/or substrates underlying the phototransduction cascade and the late OS elongation are temporarily exhausted by the first flash, such that the later flashes are unable to activate the amplification cascade and the resultant OS elongation with the same velocity.

On the other hand, the quantitative relationship between the rapid contraction and the bleach level remained unaffected by the alteration of the late OS elongation properties after the first flash. We calculated the cumulative rapid contraction by summing the rapid contraction evoked by all flashes (Fig. 4c). As predicted by the hypothesis, the relationship between the cumulative rapid contraction and the corresponding bleach levels aligned well with the voltage-dependent membrane tension model (the black curve in Fig. 4c, identical to the modeled curve in Fig. 3b).

### Rapid contraction in human rod OS in response to strong bleaching stimuli

**Fig. 5:**
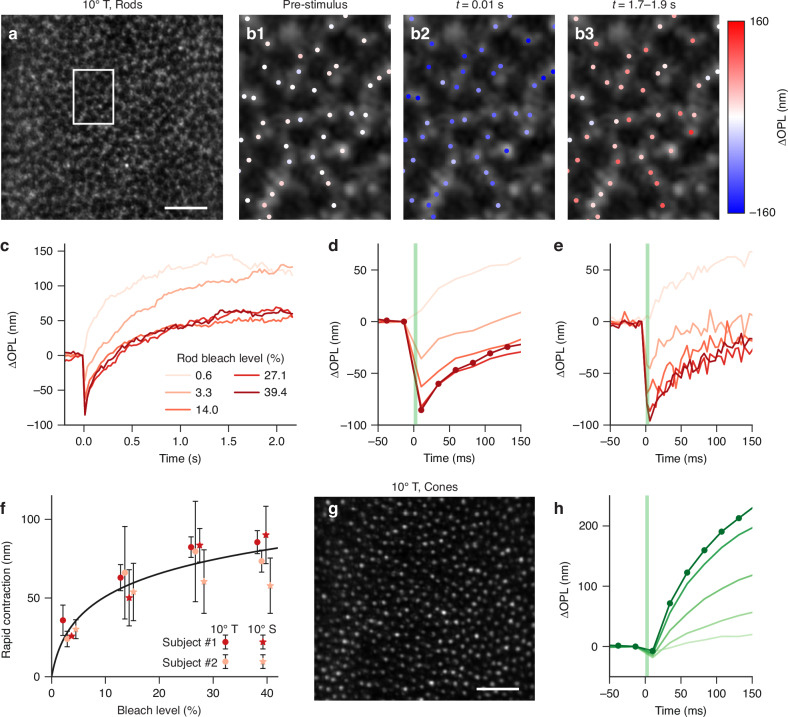
ORG responses in human rod and cone OS evoked by visual stimuli at varying strengths. **a** Maximum intensity projection at rod OS tips at 10° temporal eccentricity. Scale bar: 50 μm. **b** Enlarged view of the rod OS tips (enclosed white region in Fig. 5a). The colored dots denote manually selected locations for probing rod ORG responses, with color grading indicating the ∆OPL along the rod OS. (b1) ∆OPL extracted from the volume right before the visual stimulus, (b2) ∆OPL extracted from the volume immediately post the stimulus, indicating rapid contraction, and (b3) ∆OPL averaged over a period 1.7–1.9 s after the visual stimulus, indicating late elongation. **c** Rod OS signals in response to stimuli bleaching 0.6%-39.4% rhodopsins. **d** The enlarged view of the rod OS contraction. **e** Corresponding rod OS responses extracted using an ultrafast analysis method, where temporally sub-sampling each volume into five sub-volumes along the slow scan dimension increases the sampling rate to ~200 Hz. **f** Amplitude of rapid rod OS contraction showed a logarithmic dependence on the bleach level (black curve). The rapid contraction amplitudes were measured in two subjects at 10° temporal and 10° superior (see Fig. S8 for complete traces). The colored markers indicate the mean values, and the error bars represent the corresponding standard deviation ranges. Small jitters were introduced to the horizontal axis to reduce overlap between data. **g** Maximum intensity projection at cone OS tips from the same region as in Fig. 5a. Scale bar: 50 μm. **h** Cone OS responses extracted from the same experiment. Stimulus strengths ranged from 2.8 × 10^5^-2.7 × 10^7^ photons/μm^2^, corresponding to 0.6% (light green) to 39.4% (deep green) rhodopsin bleach (see legend in Fig. 5c and Table S4)

Next, we conducted ORG experiments in human subjects using an AO line-scan OCT system^43^ to explore whether the rapid rod OS contraction observed in rodents also occurs in humans. Rod and cone OS tips can be distinguished in depth using the system (see Fig. S7), which allows for the simultaneous analysis of rod and cone ORG signals in response to the same visual stimulus. Figure 5a–e illustrate rod ORG signals measured from the 10° temporal retina at a volume rate of 41.4 Hz. In the en-face image at the rod OS tips (Fig. 5a), rod photoreceptors can be identified (see the enlarged view in Fig. 5b). In response to a 5-ms, 470 nm stimulus that bleached 27.1% of rhodopsin, we observed consistent rapid contraction (t = 10 ms) and late elongation (t = 1.7–1.9 s) in rod OS (colored dots in Fig. 5b). Figure 5c, d show the rod OS responses, averaged across ~400 selected locations within the entire field of view (Fig. 5a), evoked by visual stimuli of varying strengths. At a 0.6% bleach level, the rod OS exhibited a typical elongation response, consistent with the previous reports^19,22^. Interestingly, at higher bleach levels, we observed a rapid contraction preceding the late elongation. To enhance the temporal resolution to ~200 Hz, we divided each volume into five sub-volumes along the slow scan dimension and extracted rod ORG signals from each sub-volume (Fig. 5e). As shown in Fig. 5e, the contraction amplitude gradually increased with bleach strength, with peak contraction occurring 2-6 ms post-stimulus. The contraction amplitude saturated at ~100 nm for bleach levels above 27.1%. On the other hand, when inspected even with the increased temporal resolution (200 Hz), the lowest bleach strength tested (0.6%) did not show an observable contraction.

Figure 5g and 5h display the en-face image at the cone OS tips and the associated ORG signals extracted from the cone OS. In comparison to rod OS (see Fig. 5d), the ORG signals from cone OS in Fig. 5h exhibited a significantly smaller contraction, followed by a much larger elongation at a higher velocity. Previous studies have attributed a substantial portion of this elongation to osmotic swelling^16,23^. The faster elongation observed in the cone OS may be related to its larger plasma membrane area^26^, which can facilitate more efficient water flow (see Discussion).

Figure 5f shows that the amplitude of rapid rod OS contraction increased logarithmically with increasing bleach levels, similar to measurements in rodent rod OS. This suggests that the rapid rod OS contraction observed in rodents and human subjects shares similar underlying mechanisms. However, there exist obvious qualitative differences between species. First, the amplitude of rod OS contraction is substantially greater in rodents for the same bleach strength. Second, the recovery of the OS contraction is significantly faster in humans. This faster recovery indicates that a time-dependent model may be required to describe the bleach-strength dependence of contraction amplitude measured in human rod OS^25^.

## Discussion

Ex vivo studies in rods have reported rapid changes in their structural and optical properties upon exposure to visual stimuli. For instance, studies in intact frog retina and individual osmotically intact rod OS have revealed a birefringence loss occurring at a rate aligned with Meta II formation^44,45^. In addition, light scattering experiments in suspensions of fragmented bovine rod OS have reported signals corresponding to the intermediate steps of phototransduction^46^. However, the models proposed to explain such changes have been inconsistent with each other^44,45,47,48^. Here, we discovered that rodent and human rod OS undergo a rapid OS contraction upon photoisomerization whose magnitude scales logarithmically with bleach strength (Figs. 3b & 5f). Additionally, we observed that the OS contraction was synchronous with an expansion of the rod IS (Figs. 2 & S4). Using serial flashes, we further confirmed that the rod OS contraction and elongation have fundamentally different kinetics and light sensitivity, such that a first priming flash can readily segregate the two components (Fig. 4). Finally, a comparison of cone and rod responses in the same human subjects and retinal locations undertaken with AO revealed substantial differences in kinetics, light sensitivity and magnitude of OS length change (contraction and expansion) between species and photoreceptor types.

While this study observed a similar rapid contraction in the rod OS in both rodents and human subjects, there were notable differences between the two species. In rodents, the contraction was significantly more persistent, and took longer to recover compared to the human rod OS. Furthermore, the secondary contraction of the rod OS, which was prominent in rodents at higher bleach levels (>11.5%), was largely absent in the human rod OS response. A few previous studies have touched on the possibility that a substantially decreased dark current could result in an osmotic imbalance that shrinks the rod OS^49,50^. However, the precise mechanisms underlying the secondary contraction in rodents and the observed discrepancies between rodent and human rod OS responses remain to be elucidated. To better understand these differences, future studies may investigate the potential influence of dark adaptation protocols (12 hours in rodents versus 15 minutes in human subjects) on the rod OS response. Broadly, comparative studies exploring the structural and physiological differences in rods among various species may provide valuable insights into the factors contributing to these differences.

In human subjects, the amplitude of rapid contraction in both rod (see Fig. 5f) and cone OS saturated logarithmically with increasing bleach levels^13^. However, the maximum contraction amplitude measured in rod OS reached up to ~100 nm, which is significantly larger than the ~50 nm measured in cone OS^13^. One notable difference between primate cone and rod photoreceptors is that the length of rod OS is significantly greater than that of cone OS^51,52^. The accumulation of disk membrane contraction will thus be greater along a longer OS in rods compared to cones. Additionally, the ultrafast analysis shown in Fig. 5e revealed that, in response to strong bleaching stimuli, the human rod OS elongated by ~40 nm within the first 50 ms following the rapid contraction, corresponding to a maximum, saturated elongation velocity of 0.8 $\text{nm}/\text{ms}$. In contrast, the maximum elongation velocity of cone OS is significantly faster (~4 $\text{nm}/\text{ms}$)^13,16^. This difference aligns with the distinct membranous organization of rod and cone OS. In cone OS, the infoldings of the plasma membrane create a larger surface area that facilitates more efficient water transport across the membrane, whereas in rod OS, the disks are enclosed within a tightly fitting cylindrical membrane^26^. This difference in the velocities of late elongation may also contribute to the apparent difference in the maximum contraction amplitudes between human rods and cones.

Although ERP signal has been recorded from both rods and cones^28^, it is important to note that in rods, only a small fraction of rhodopsins (estimated to be 2% of the total rhodopsins in mouse rods^29^) present in the plasma membrane and the small number of nascent, basal disks can contribute to the measured electrical signal^28^. As such, despite the abundance of rhodopsin in the human and monkey retina, the ERP measured in these species using electrical approaches was primarily from the activation of cone opsins^53,54^. Remarkably, the rapid rod OS contraction reported in this study is likely to reflect the cumulative contraction of individual sealed disks, which are electrically uncoupled from the plasma membrane and thus difficult to access in electrophysiology^27,28^. The measured bleach-strength dependence of the contraction aligned with the model prediction based on the voltage-dependent membrane tension during ERP, whether assessed by a single flash (Fig. 3b) or a serial bleach protocol (Fig. 4c). The latter has been widely used in studies of the ERP, where an exponential decay in the electrical potential was observed due to a similar exponential reduction in the rhodopsin concentration following preceding flashes^28,29,34^. Unlike the linear relationship noted between the ERP and bleached pigment in prior studies^33,34^, the rod OS contraction exhibits a logarithmic saturation as described well by the voltage-dependent membrane tension accompanying electrical activity.

Overall, label-free optical imaging of the rapid rod OS dynamics in vivo can uncover the first steps in rhodopsin activation that were previously challenging to access, even in electrical recordings. Importantly, the objective, non-invasive, and spatially-localized monitoring of rod photoreceptor function may enable the sensitive assessment of impaired dark adaptation and visual retinoid cycle in retinal diseases.

## Materials and methods

### Ultrahigh-resolution point-scan OCT system for rodent retina imaging

**Fig. 6:**
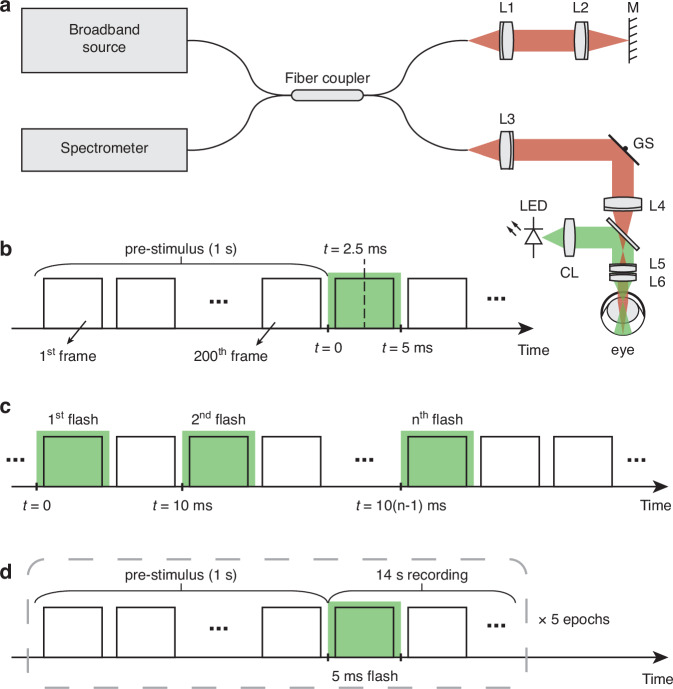
System setup and the timing diagrams for data acquisition and visual stimulation. **a** A spectral-domain point-scan OCT system (red path) was integrated with a visual stimulation channel (green path). L1-L6: doublet lenses. CL: condenser lens. GS: galvo scanner. M: mirror. **b** Protocol I: a 5-ms flash was delivered 1 s after the start of the OCT recording. **c** Protocol II: multiple 5-ms flashes were delivered at time points of 0, 10 ms, …, 10(n-1) ms, where n is the total number of flashes. **d** Protocol III: five epochs, each consisting of a 15-s recording, were captured consecutively. A 5-ms visual stimulus was delivered 1 s after the start of each epoch

The ORG experiments were performed using a custom-built spectral-domain ultrahigh-resolution OCT system (Fig. 6a)^22^. The system employed a superluminescent diode (cBLMD-T-850-HP-I, Superlum, Ireland) with a center wavelength of 840 nm and a full-width at half-maximum (FWHM) of 146 nm, providing a theoretical axial resolution of 2.0 μm in tissue. The OCT beam power at the cornea was measured to be 2.0 mW, resulting in a rhodopsin bleaching rate of 0.02% per second (see Supplementary Section 5.3), which is much weaker than the bleaching caused by the 5-ms visual stimulus used in ORG measurements ($\ge$2.3%). A spectrometer equipped with a line-scan camera (CS800-800/300-250-OC2K-CL, Wasatch Photonics, USA) captured spectral interferograms at a line-scan rate of 250 kHz and allowed an imaging depth of 1.1 mm in air. An afocal telescope, consisting of a scan lens (80 mm doublet) and an ocular lens (30 mm + 25 mm doublets), conjugated the galvo scanner and the pupil plane with a magnification of 0.17. This arrangement reduced the OCT beam size and enlarged the scan angle. Based on a standard rat eye model^55^, the theoretical diffraction-limited beam size on the retina was estimated to be 7.2 μm (FWHM). AO was not utilized in rodent imaging because the improvement in lateral resolution would likely have minor benefits for extracting rod ORG signals from rodent retinas, where ~97% of photoreceptors are rods^51^. A non-AO configuration also simplified the system and demonstrated the broad applicability of the proposed approach.

A green LED (M505L4, Thorlabs, USA) was collimated by an aspheric condenser lens to provide the visual stimulation. The ocular lens converged the collimated beam and achieved a 5.82 ${\text{mm}}^{2}$ Maxwellian illumination on the posterior eye. The visual stimulus settings were converted into bleach levels based on the previously published method^56,57^, as detailed in Supplementary Section 5.1. A function generator (PCIe-6363, National Instruments, USA) synchronized the camera acquisition, galvanometer scanning, and visual stimulation. Data acquisition was controlled using an interface software developed in NI LabVIEW (19.0.1, National Instruments, USA).

### Animal preparation and ORG imaging

All experiments were conducted in compliance with guidelines and approvals from the Institutional Animal Care and Use Committee (IACUC), SingHealth (2020/SHS/1574). Wild-type Brown Norway rats (*N* = 13) were used in the study, and all imaging sessions were performed when they were between 7 and 17 weeks of age. The animals were dark-adapted overnight before each ORG experiment, and the entire experiment was conducted in a darkroom. During the experiment, animals were anesthetized with a ketamine and xylazine cocktail and secured in a custom-built stereotaxic stage to minimize eye motion. Before imaging, one drop of 1% Tropicamide (Alcon, Geneva, Switzerland) and one drop of 2.5% Phenylephrine (Alcon, Geneva, Switzerland) were applied to the eye to dilate the pupil. The cornea was kept moist during the imaging by frequently applying a balanced salt solution.

Repeated cross-sectional scans, each consisting of 1000 A-lines acquired in 4 ms, were collected from the retina. We investigated ORG signals evoked by a single flash at varying bleach levels (2.3–62.6%, Protocol I), and ORG signals in response to serial flashes with (Protocol II) short inter-flash intervals and (Protocol III) long inter-flash intervals. Note that to avoid interference from preceding bleaching, each eye was used for only one acquisition per imaging session.

In Protocol I, a 5-ms visual stimulus was delivered to the retina 1 s after the start of the OCT recording (see Fig. 6b). To capture the rapid dynamics evoked by the flash, the first 600 B-scans were acquired at a temporal resolution of 5 ms (4 ms acquisition + 1 ms galvo flyback time, i.e., a duty cycle of 0.8) for 3 s. To record the prolonged dynamics while reducing computational load, we continued the recording with 1400 B-scans at a temporal resolution of 25 ms (4 ms acquisition + 21 ms galvo flyback time, i.e., a duty cycle of 0.16) for 35 s. The total recording time was 38 s, including a 1-s pre-stimulus period. In all experiments, the onset of the first flash was set as time point 0.

Protocol II used the same OCT recording configuration as Protocol I, but instead of a single flash, multiple 5-ms flashes were delivered 1 s after the start of the OCT acquisition (see Fig. 6c). The flashes were delivered at time points of 0, 10 ms, …, 10(n-1) ms, where n is the total number of flashes.

In Protocol III, the entire recording consisted of five consecutive epochs (see Fig. 6d). Each epoch comprised 600 B-scans at a higher temporal resolution (5 ms) for 3 s and 480 B-scans at a lower temporal resolution (25 ms) for 12 s. A 5-ms visual stimulus was delivered 1 s after the start of each epoch.

### Preprocessing of OCT images and extraction of phase traces for ORG measurements in rodents

The raw spectral interferograms first underwent spectral calibration and k-linearity. Then, a discrete Fourier transform (DFT) was applied to generate complex-valued OCT images. To minimize signal decorrelation caused by bulk tissue motion, we used our proposed phase-restoring subpixel image registration method to register complex-valued OCT images with subpixel precision^58^. Specifically, the subpixel-level translational displacements between subsequent frames and the first frame were determined using the single-step DFT algorithm^59^. Then, the axial and lateral displacements were corrected by multiplying exponential terms in the spectral and spatial frequency domain, respectively.

To detect light-evoked retinal dynamics, we calculated the temporal phase change between two retinal layers. First, we removed the arbitrary phase offset by calculating the phase change with respect to the first B-scan,

3 $$\widetilde{{I}^{{\prime} }}({s},{i})=\widetilde{I}({s},{i})\exp [-j{\text{arg}}(\widetilde{I}({s},1))]$$

where $\widetilde{I}\left(s,i\right)$ represents the complex-valued OCT signal after image registration. $s$ and $i$ denote the spatial and temporal indices, respectively. $j$ is the imaginary unit. $\text{arg}\left(\,\right)$ denotes the calculation of the argument. $\widetilde{I^{\prime}}\left(s,i\right)$ is the corresponding time-referenced signal.

We further performed self-referenced measurements to remove residual bulk phase error, resulting in phase differences between two retinal layers. Specifically, for each pixel of interest in the target layer, referred to as ${s}_{t}$, its temporal phase change was calculated relative to a reference region chosen from a reference layer. The reference region was centered on the same A-line as the pixel of interest and spanned across 5 adjacent A-lines. The phase change of the reference region, ${\varphi }^{r}$, was obtained by spatially averaging the time referenced OCT signals and extracting the phase component,

4 $${\varphi }^{r}\left({s}_{t},\,i\right)={\text{arg}}\left(\frac{1}{N}{\sum }_{s={s}_{r1}}^{{s}_{{rN}}}\widetilde{{I}^{{\prime} }}\left(s,i\right)\right)$$

where ${s}_{r1}$, ${s}_{r2}$, …, ${s}_{{rN}}$ represent the spatial index of individual pixels in the selected reference region, and $N$ is the total number of pixels in the reference region.

By referring to the reference region, the phase change of the pixel ${s}_{t}$, denoted as ${\varphi }_{{tar}/{ref}}\left({s}_{t},{i}\right)$, can be calculated as follows,

5 $${\varphi }_{{tar}/{ref}}\left({s}_{t},\,i\right)={\text{arg}}\{\widetilde{{I}^{{\prime} }}\left({s}_{t},i\right)\exp \left[-j{\varphi }^{r}\left({s}_{t},\,i\right)\right]\}$$

We applied the same process to every pixel in the target layer to obtain spatially resolved phase traces. Traces with a pre-stimulus standard deviation less than 0.5 radians were retained for further analysis. Note that to enhance the signal-to-noise ratio (SNR), phase traces extracted from individual B-scans spanning over 4 ms were spatially averaged within each signal pattern based on the Knox-Thompson algorithm^60^. We assigned the midpoint of the 4-ms recording as the timestamp for the averaged phase traces extracted from the corresponding B-scan. For example, the first B-scan recorded after the flash onset at 0 ms spanned from 0.5 ms to 4.5 ms, and the corresponding averaged phase traces were assigned a timestamp of 2.5 ms (see Fig. 6b). The obtained phase change can be further converted into ∆OPL by multiplying it by ${\lambda }_{c}/4\pi$, where ${\lambda }_{c}$ is the center wavelength of the OCT system.

### ORG experiments in human subjects using an AO line-scan OCT system

Two male subjects free of retinal disease were enrolled in this study. All subjects provided written informed consent before participating. An AO line-scan OCT system, as described in a previous study^16,43^, was used for human ORG experiments. The OCT beam power at the cornea was 1.5 mW, causing minor rhodopsin bleaching ( ~ ${10}^{-6}$ per second, see Supplementary Section 5.3) compared to the bleaching induced by the visual stimuli used in ORG recordings ($\ge$0.6%). The experimental protocol was approved by the University of Washington Institutional Review Board, and all procedures were in accordance with the tenets of the Declaration of Helsinki.

Cycloplegia was introduced using tropicamide 1% ophthalmic solution (Akorn Inc.) before OCT imaging to dilate the pupil. The subjects underwent a 15-minute dark adaptation before each imaging session that replenished up to 90% of rhodopsin^11^. Each session consisted of two recordings: one centered at 10° temporal and the other at 10° superior. The visual stimulation field was sufficiently small to ensure no overlap between the two regions. Two experimental protocols were employed:

*Protocol 1*: 100 volumes, each consisting of 500 B-scans and covering a 0.8°$\times$1° field of view (FOV) were recorded at a volumetric rate of 41.4 Hz.

*Protocol 2*: 50 volumes, each consisting of 600 B-scans and covering a FOV of 0.92°$\times$1°, were recorded at a volumetric rate of 17.3 Hz.

In both recording protocols, a 5-ms visual stimulus generated by a 470 nm LED was delivered in Maxwellian view at the start of the 11th volumetric scan. Stimulus parameters were converted into bleach levels, as detailed in the Supplementary Section 5.2. The radiant exposure safety assessment in Supplementary Section 5.4 shows that the highest radiant exposure ( < $1\times {10}^{-2}\,\text{J}/{\text{cm}}^{2}$) is well below the permissible limit (0.8 $\text{J}/{\text{cm}}^{2}$). Each visual stimulus condition was repeated five times to enhance the SNR of the measured ORG.

### Extraction of ORG signals in human ORG measurements

OCT data were processed using conventional techniques as described previously^16,43^. The acquired interference fringe data were resampled in k-space and Fourier-transformed along the wavenumber dimension, resulting in complex-valued OCT volumes. Each retinal volume was segmented, and en-face images were generated by taking maximum-intensity projections centered at the cone OS tips. En-face images were registered using a strip-based registration algorithm^61^, with lateral shifts from registration and axial shifts from segmentation being applied to register all volumes. Cones and rods were manually selected based on the maximum intensity projections of the cone OS tips and rod OS tips, respectively.

To measure ORG signals, we first referenced registered OCT volumes to the mean of all pre-stimulus volumes to remove arbitrary phase offsets. Next, we multiplied OCT signals at the photoreceptor OS tips with the complex conjugate of OCT signals at the IS/OS. The argument of the resulting product yielded temporal phase changes between the two boundaries of the photoreceptor OS. We computed ORG responses by integrating phase across a 3$\times$3 pixel square centered at manually selected locations and averaging them across up to five repeated image acquisitions. For phase responses exceeding $\pm \pi$ radians, the phase was unwrapped along the temporal dimension. The phase change was converted into ∆OPL by multiplying it by ${\lambda }_{c}/4\pi$, where ${\lambda }_{c}$ is the center wavelength of the OCT system, equal to 820 nm.

To explore the ORG responses at a higher temporal resolution, we leveraged the scan dimension as a secondary, higher-resolution time axis^13^. The entire volume was divided into five sub-volumes along the scan direction. ORG responses were extracted from rods within each sub-volume and averaged to obtain one value for each sub-volume. We assigned the midpoint of the scan-time across individual sub-volumes as the timestamp of the averaged ORG responses. Therefore, a 41.4 Hz volume rate was effectively increased 5-fold in its temporal sampling to reveal features that may otherwise be obfuscated.

## Supplementary information

Supplementary Information

## Data Availability

All data needed to evaluate the conclusions in the paper are present in the paper. Additional data related to this paper may be requested from the authors.
