# Supplementary material for: Optoretinography reveals rapid rod photoreceptor movement upon rhodopsin activation
Source: Light Sci Appl. 2026 Jan 7;15:58. doi: 10.1038/s41377-025-02149-6 (PMC12775420; doi:10.1038/s41377-025-02149-6)
Supplement: Supplementary file 1 — Supplementary Information [file 41377_2025_2149_MOESM1_ESM.pdf]

Supplementary Information for

Optoretinography reveals rapid rod photoreceptor movement upon rhodopsin activation

Huakun Li[1], Connor E. Weiss[†2,3], Vimal Prabhu Pandiyan[†2,4], Davide Nanni[†1], Teng Liu[2,5], Pei Wen Kung[6], Bingyao Tan[6,7,8], Veluchamy Amutha Barathi[6,8,9], Leopold Schmetterer[1,6,7,8,10,11,12,13], Ramkumar Sabesan*[2,4], Tong Ling*[1,6,7,14]

[1]*School of Chemistry, Chemical Engineering and Biotechnology, Nanyang Technological University, Singapore, Singapore.*

[2]*Department of Ophthalmology, University of Washington School of Medicine, Seattle, WA 98109, USA.*

[3]*Graduate Program in Neuroscience, University of Washington, Seattle, WA 98109, USA.*

[4]*Roger and Angie Karalis Johnson Retina Center, Seattle, WA, USA.*

[5]*Department of Bioengineering, University of Washington, Seattle, WA 98109, USA.*

[6]*Singapore Eye Research Institute, Singapore National Eye Centre, Singapore, Singapore.*

[7]*SERI-NTU Advanced Ocular Engineering (STANCE) Program, Singapore, Singapore.*

[8]*Ophthalmology & Visual Sciences Academic Clinical Program (EYE ACP), Duke-NUS Graduate Medical School, Singapore, Singapore.*

[9]*Department of Ophthalmology, Yong Loo Lin School of Medicine, National University of Singapore and National University Health System, Singapore, Singapore.*

[10]*AIER hospital group, Changsha, China.*

[11]*Department of Clinical Pharmacology, Medical University of Vienna, Vienna, Austria.*

[12]*Center for Medical Physics and Biomedical Engineering, Medical University of Vienna, Vienna, Austria.*

[13]*Rothschild Foundation Hospital, Paris, France.*

[14]*School of Electrical and Electronic Engineering, Nanyang Technological University, Singapore, Singapore.*

† These authors contributed equally

* Correspondence should be addressed to: rsabesan@uw.edu, tong.ling@ntu.edu.sg

Supplementary Section 1: Separating the light-evoked dynamics of rod OS tips and the RPE-BrM complex using unsupervised learning

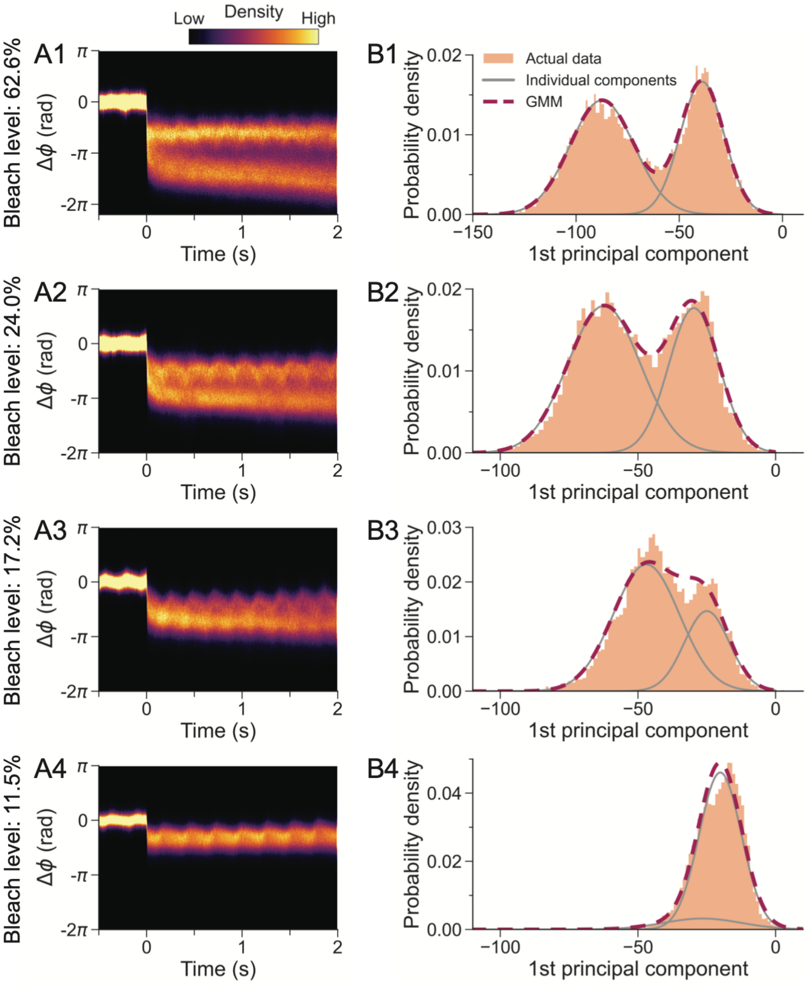

Figure S1. Distributions of phase traces evoked by bleaching stimuli at varying bleach levels. (A) Distribution density maps of phase traces extracted from the composite layer using the inner segment/outer segment junction as the reference. (B) Principal component analysis was independently conducted on each dataset, and phase traces were projected onto the corresponding 1st principal component. For each dataset, the distribution of the principal component score (light brown bins) was fitted with a Gaussian mixture model consisting of two components (GMM, dashed dark magenta line). The gray curves represent two individual components.

As shown in Fig. 1C and Fig. S1, the two ORG signal patterns that are readily distinguishable at high bleach levels begin to overlay at lower bleach levels, making temporal features alone less effective in separating them. To consistently separate the two light-evoked dynamic patterns across measurements at different stimulus strengths, we analyzed the distribution of two signal patterns in space. For each data point in Fig. 1C, we retrieved its spatial location, calculated the distance to the bottom of the BrM, and further obtained a normalized distance that was divided by the mean thickness of the composite layer. Figure S2A illustrates the distribution of the normalized distances for the two types of signals. Based on this depth distribution profile, Type-I corresponds to rod OS tips, while Type-II can be attributed to the RPE-BrM complex[1]. We explored optimal decision boundaries for isolating these retinal structures based on the F_β score, which is defined as,

$$F_\beta = (1+\beta^2)\cdot\frac{\text{precision}\cdot\text{recall}}{\beta^2\cdot\text{precision}+\text{recall}} \tag{S1}$$

$$\text{precision} = \frac{TP}{TP+FP} \tag{S2}$$

$$\text{recall} = \frac{TP}{TP+FN} \tag{S3}$$

where TP, FN, and FP represent the number of true positives, false negatives, and false positives, respectively.

In this study, we set β to 0.5 to place more emphasis on precision over recall. Figure S2B shows the $F_{0.5}$ scores calculated for both signal types at varying thresholds. For each signal type, the optimal threshold was determined based on the depth that achieves the maximum $F_{0.5}$ score (see red and blue stars in Fig. S2B). The associated normalized confusion matrices were shown in Figs. S2C-D. Accordingly, the composite layer can be divided into rod OS tips, a transition region, and the RPE-BrM complex.

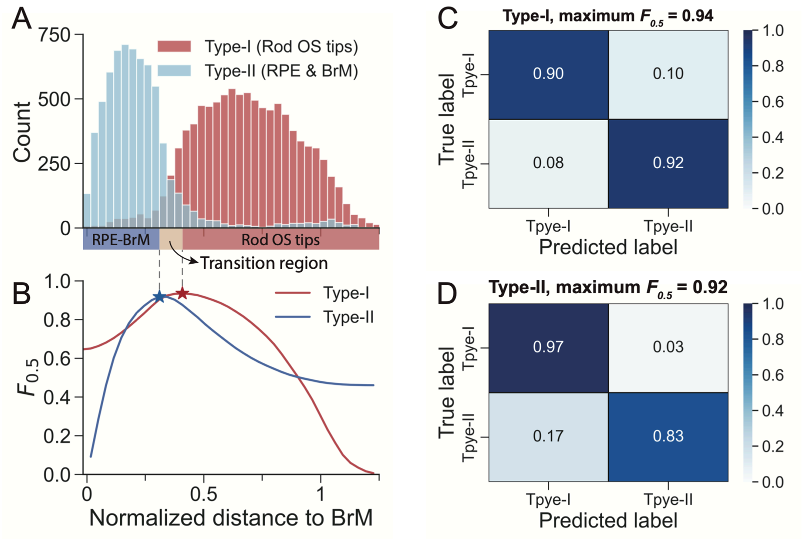

Figure S2. Clustering of two signal patterns based on the axial distance to Bruch's membrane (BrM). (A) The depth distribution of signals shown in Fig. 1C. For each pixel, the distance to BrM was calculated and normalized by dividing the average thickness of the composite layer. (B) $F_{0.5}$ scores for the Type-I signal (red curve) and Type-II signal (blue curve) at varying depth thresholds. Star markers indicate the thresholds with the highest $F_{0.5}$ scores. Normalized confusion matrices at the thresholds indicated by the (C) red star and (D) blue star in panel B.

Supplementary Section 2: Delineating the relative contribution of refractive index and mechanical movements in the rapid OS contraction

The observed rapid OPL decrease in the OS may stem from changes in the refractive index, the physical length, or a combination of both. Thus, it is essential to determine the relative contributions of both factors to the observed OS contraction. With respect to refractive index changes, it can arise in the rod OS 1) from a loss in visual pigment-related anomalous dispersion following rhodopsin activation. Refractive index and absorption are interconnected through the Kramers-Kronig relations, implying that visual pigment bleaching can lead to changes in the refractive index[2]. However, as shown in Fig. S3, this effect associated with rhodopsin bleaching was found to be minor at our OCT imaging wavelengths (750 nm-950 nm)[3,4]. 2) Refractive index changes may also occur during the reorientation of anisotropic molecules within the disk membranes and alterations in the arrangement of lamellar dielectric structures that comprise disk membranes, cytoplasmic and intradisk spaces[4,5]. The former would result in intrinsic birefringence changes, whereas the latter can lead to variations in form birefringence. As a reference for their scales, rhodopsin activation induced a decrease in birefringence of 7×10^{-5} in osmotically intact frog rod OS at wavelengths longer than 650 nm[4], which can only account for an OPL change of a few nanometers, substantially smaller than the > 100s of nm rod OS contraction shown here.

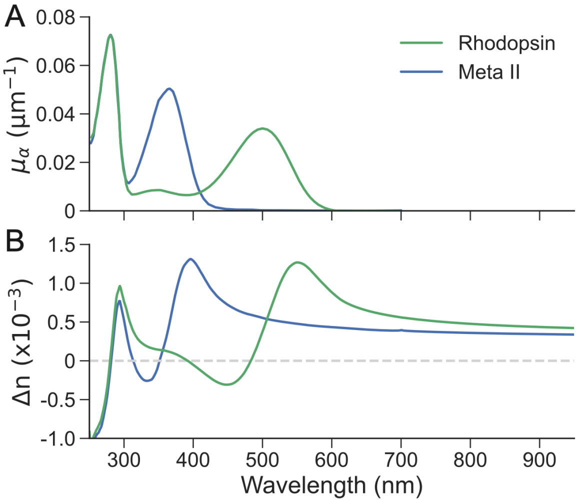

Figure S3. Modulation of refractive index calculated using the Kramers-Kronig relation. (A) Absorption spectra of rhodopsin and metarhodopsin II (Meta II)[6]. The rhodopsin absorption at 498 nm was measured to be ~0.015 optical density (OD) per micrometer[7], corresponding to an absorption coefficient μ_α of 0.034 µm^{-1}. (B) Wavelength-dependent variations in refractive index (Δn) derived from the Kramers-Kronig relation[2]. At the OCT center wavelength (840 nm), the Δn values are 4.57×10^{-4} for rhodopsin and 3.52×10^{-4} for Meta II. This decrease in refractive index upon rhodopsin bleaching (approximately -1×10^{-4}) can only account for an optical path length (OPL) decrease of ~2.4 nm along a 24-µm rod outer segment.

Another key evidence for differentiating the contribution of physical length change from that of the refractive index change is the rapid OPL changes in adjacent retinal layers (see Fig. 2A), including the IS and the space between OS tips and the RPE-BrM complex. These OPL changes cannot be attributed to refractive index changes within the OS where rhodopsins are activated. A plausible interpretation for the rapid OPL changes in adjacent

retinal layers is that the OS undergoes an intrinsic mechanical shrinkage that reduces the ΔOPL in the OS; such a movement would pull the IS posteriorly via the IS/OS junction and the RPE-BrM complex anteriorly via connected microvilli[8,9]. This interpretation is consistent with the rapid OPL increase in the IS and the smaller magnitude of contraction in the SRS (between the ELM and RPE/BrM) compared to that between the ELM and OS tips. Furthermore, if the refractive index change associated with photoisomerization is the primary origin of the rapid OPL decrease in the OS, the amplitude of the OPL decrease is expected to scale linearly with the bleach level, which doesn't align with the observed nonlinear dependence of the rapid OPL decrease on the bleach level (see Fig. 2A).

Taken together, these experimental findings suggest that mechanical movements, instead of changes in refractive index, play a dominant role in the rapid OPL changes observed upon rhodopsin activation in this study.

Supplementary Section 3: Testing whether the rapid OS shrinkage can be explained based on the osmolarity-driven water efflux model

If the rapid shrinkage of the rod OS is driven by a sudden decrease in osmotic pressure, the water permeability coefficient of the rod OS plasma membrane can be estimated based on a model developed by Zhang *et al.*[10], with parameters listed in Table S1. Assuming that the shrinkage is purely an osmotic effect, Van't Hoff's law suggests that the saturated decrease in cytoplasmic volume, denoted as ΔV_{cyto}, is proportional to the initial decrease in the osmotic pressure,

$$\Delta V_{\text{cyto}}/V_{\text{cyto, rest}} = \Delta\Pi/\Pi_{\text{rest}} \tag{S4}$$

where $V_{\text{cyto, rest}}$ and Π_{rest} represent the cytoplasmic volume and osmotic pressure of the rod OS in the rest (dark-adapted) state. $\Delta\Pi$ is the change in the osmatic pressure triggered by visual stimuli. Since moderate osmotic changes do not affect the width of the rod OS[10] and the cytoplasmic space occupies about 44% of the space within the rod OS[11], the fractional volume decrease $\Delta V_{\text{cyto}}/V_{\text{cyto, rest}}$ can be calculated as $\Delta L_{\text{ROS}}/(0.44 \times L_{\text{ROS}})$. In response to a flash at 62.6% bleach, the change in cytoplasmic pressure can be calculated by $\Delta\Pi/RT = [\Delta L_{\text{ROS}}/(0.44 \times L_{\text{ROS}})] \cdot (\Pi_{\text{rest}}/RT) = -4.39$ mOsM.

The water flow rate depends on the osmotic pressure difference across the membrane and the hydraulic conductivity L_p of the plasma membrane. The initial water flow rate following a sudden decrease in the osmotic pressure, denoted as J_w, can be calculated by,

$$J_w = L_p S_{\text{ROS}} \Delta\Pi \tag{S5}$$

In our measurements, we found that the rapid contraction saturated 10 ms after the flash onset. Since the water flow rate driven by a sudden osmolarity change would gradually decrease as it approaches a new osmotic equilibrium, a conservative estimation of the initial flow rate is $\Delta L_{\text{ROS}}/10\text{ ms} = -14.26$ µm s^{-1} , or -32.37 µm^3 s^{-1} with a cross-sectional area of 2.27 µm^2. According to Eq. (S5), the hydraulic conductivity L_p can be calculated as 216.78 µm^3 s^{-1} dyn^{-1}, and the corresponding water permeability coefficient is 0.30 cm s^{-1}. This value is much larger than 2.6×10^{-3} cm s^{-1} measured in previous in-vitro studies[12]. This discrepancy suggests that the observed rapid shrinkage of the rod OS is too fast to be attributed to osmosis.

Table S1. Parameters used for testing the osmolarity-driven water efflux model.

Parameter	Value	Unit
Normal rodent plasma osmolarity[13], Π_{rest}/RT	325	mOsM
Length of the rod OS[14], L_{ROS}	24.0	µm
Maximum contraction*, ΔL_{ROS}	-142.6	nm
Surface area of the rod outer segment, S_{ROS}	132	µm^2
Cross-sectional area of the rod OS[14], A_{ROS}	2.27	µm^2
Initial water influx rate*, J_w	-32.37	µm^3 s^{-1}

* Calculated with a refractive index of 1.41.

Supplementary Section 4: Contraction of the rod OS based on the voltage-dependent membrane tension model

The disk membranes in cones are confluent with the plasma membrane, while most rod disk membranes, except for a few nascent basal disks, are entirely enclosed by the plasma membrane[15,16]. Despite the distinct membranous organization of cone and rod OS[17], the following discussion provides justifications for applying the electromechanical coupling model (Eq. 2), which was established to explain the rapid contraction in the cone OS, to characterize the contraction in the rod OS.

1) The electromechanical coupling model proposed for cone early receptor potential (ERP) primarily concerns the shape changes of individual disks, which would apply to the disks in rods as well. Although rods and cones are drastically different in their membrane organization, they can share similar changes in voltage-dependent membrane tension during the ERP in individual disks. Such a driving force of voltage-dependent membrane tension can be used to estimate the steady-state equilibrium of individual disk area expansion/contraction without considering other properties of the rod OS, such as the viscosity of the cytoplasm, that may affect the time-dependent dynamics.

2) The slow recovery from rapid contraction enables modeling the rod ERP quasi-statically. In response to strong bleaching stimuli, measured ΔOPL in rodent rod OS exhibited a persistent contraction. The slow recovery rate makes the quasi-static model suitable for calculating the amplitude of the rapid contraction response in the rod OS.

Given the above considerations, we adopted the quasi-static electromechanical coupling model based on the voltage-dependent membrane tension during the ERP[18] (see Eq. 2) to model the rapid rod OS contraction, particularly its amplitude, with parameters listed in Table 1 and Table S2.

To link the area expansion in Eq. 2 with the measured ΔOPL, we assume a constant volume of individual disks during the millisecond-scale dynamics. The axial shrinkage of each disk caused by its area expansion can thus be estimated by[18],

$$\Delta z = -\frac{A(\tilde{\tau})-A(\tilde{\tau}_{\mathrm{rest}})}{A(\tilde{\tau}_{\mathrm{rest}})} z \tag{S6}$$

For the entire photoreceptor OS, we can calculate ΔOPL as,

$$\Delta \mathrm{OPL} = \Delta z \times N_{\mathrm{disks}} \times n_{\mathrm{index}} \tag{S7}$$

where N_{disks} is the number of disks along each OS, n_{index} is the refractive index.

Table S2. Parameters used for calculating the rod OS contraction based on the voltage-dependent membrane tension model.

Parameter	Value	Unit
Length of the rod OS[14], L_{ROS}	24.0	μm
Cross-sectional area of disk face in the resting state[14], $A(\tilde{\tau}_{\mathrm{rest}})$	2.1	$\mu\mathrm{m}^2$
Disk-disk distance[11], d	32	nm
Number of disks per rod, $N_{\mathrm{disks}} = L_{\mathrm{ROS}}/d$	750	/
Height of a single disk in the resting state[11], z	18	nm
Rhodopsin density on the disk membrane[19], σ_{rho}	23000	molecules $\mu\mathrm{m}^{-2}$
Charge displacement per photoisomerization*, q	0.14	electron
Specific membrane capacitance[20,21], c_m	1	$\mu\mathrm{F\ cm}^{-2}$
Area expansion modulus[18], K_A	0.2	$\mathrm{N\ m}^{-1}$
Tension/voltage[18,22], α	-0.1	$\mathrm{mN\ m}^{-1}\ \mathrm{V}^{-1}$

* Charge displacement per photoisomerization was estimated as follows:

Previous studies have suggested that a full bleach results in the displacement of 2×10^5 electronic charges across the rod plasma membrane[21], or equivalently 6.2×10^4 electronic charges shift at a 30% bleach level[23]. Considering that each rod contains 7×10^7 rhodopsin molecules[19], and 2% of rhodopsin molecules are located in the plasma membrane and nascent basal disks[24], the charge displacement per photoisomerization can be calculated as $2 \times 10^5/(7 \times 10^7 \times 2\%) = 0.14$ electrons.

Supplementary Section 5: Calculation of bleach levels and assessment of radiant exposure safety

5.1 Rhodopsin bleaching in rodents

Given the negligible regeneration of rhodopsin during visual stimulation, rhodopsin bleaching can be calculated by[7,25],

$$p(p_0, Q) = p_0 \exp(-Q/Q_e) \tag{S8}$$

$$\text{Bleach level} = p_0 - p(p_0, Q) = p_0[1 - \exp(-Q/Q_e)] \tag{S9}$$

where p_0 and $p(p_0, Q)$ represent the fraction of rhodopsin present before and after visual stimulation, respectively. We set p_0 to 1 when calculating the rhodopsin bleaching in rats, as the animals were dark-adapted overnight before the ORG experiments[25]. Q is the effective energy density (photons μm^{-2}) of the visual stimulus, which can be obtained by[7],

$$Q = \frac{t_f}{A} \cdot \int_0^{+\infty} \frac{P(\lambda)S(\lambda)}{E_v(\lambda)} d\lambda \tag{S10}$$

where t_f is the duration of the flash, A is the illuminated area on the retina, $P(\lambda)$ is the power spectral density of the visual stimulus (W nm^{-1}), $S(\lambda)$ is the normalized absorption spectrum of the visual pigment calculated based on the Lamb template[26]. $E_v(\lambda)$ is the energy of a single photon at wavelength λ and can be calculated by hc/λ, where h and c represent the Planck constant and the speed of light in vacuum, respectively. Q_e is the energy density that reduces rhodopsin to $1/\mathrm{e}$ of its dark-adapted level, which was measured to be 7.94×10^7 photons μm^{-2} in rats[25]. Note that both Q and Q_e are calculated by dividing the energy measured at the cornea by the illumination area on the retina[7]. The bleach levels of flashes used in rodent ORG experiments are summarized in Table S3.

Table S3. Stimulus parameters in rodent ORG experiments.

**Energy density Q (photons μm^{-2})**	**Bleach level (%)**
1.88×10^6	2.3
2.83×10^6	3.5
4.27×10^6	5.2
6.72×10^6	8.1
9.75×10^6	11.5
1.50×10^7	17.2
2.17×10^7	24.0
3.30×10^7	34.0
5.04×10^7	47.0
7.81×10^7	62.6

5.2 Calculation of bleach levels in human subjects

The bleach level in human rods can be calculated by[27,28],

$$\text{Bleach level} = p_0\left[1 - \exp\left(-Q_{\text{retina}}/Q_{e,\text{retina}}\right)\right] \tag{S11}$$

where Q_{retina} is the energy density of the flash stimulus at the retinal surface, which can be calculated by,

$$Q_{\text{retina}} = \frac{t_f}{A} \cdot \int_0^{+\infty} \frac{\tau(\lambda)P(\lambda)S(\lambda)}{E_v(\lambda)} d\lambda \tag{S12}$$

Note that compared to Eq. (S10), the transmissivity of the ocular media[29], denoted by $\tau(\lambda)$, is incorporated into the calculation in Eq. (S12).

For rhodopsin bleaching, $Q_{e,\text{retina}}$, which represents the energy density required to reduce rhodopsin to $1/\text{e}$ of its dark-adapted level, can be calculated by,

$$Q_{e,\text{retina}} = 1/f_{\text{Dens}} f_{wg} \alpha_{\lambda_{\text{max}}} \gamma \tag{S13}$$

where f_{Dens} is a factor accounting for the "self-screening" effect, i.e., the decrease in the power along the length of the dark-adapted rod OS[27,28]. f_{Dens} has been calculated to be $1/1.44$, given an axial density of 0.45 in the human rod OS[27]. $f_{wg} \alpha_{\lambda_{\text{max}}} \gamma$ represents rhodopsin photosensitivity and has been measured to be 3.1×10^{-8} μm^2 photon^{-1} using a scanning laser ophthalmoscope[27] in humans.

The fraction of rhodopsin present after a 15-min dark adaptation, can be calculated according to the "MLP rate-limited kinetic model"[27],

$$p_0 = 1 - K_m W\left\{\frac{B_0}{K_m} \exp\left(\frac{B_0}{K_m}\right) \exp\left(-\frac{1+K_m}{K_m} rt\right)\right\} \tag{S14}$$

where $W(x)$ is the "Lambert W" function[30]. B_0 represents the fraction of rhodopsin bleached before dark adaptation. The parameters K_m and r were 0.25 and 0.8 min^{-1}, respectively, based on the time course of rhodopsin regeneration measured in normal human subjects[27]. Using $B_0 = 1$ and $t = 15$ min, we obtained $p_0 = 0.91$, indicating that a 15-min dark adaptation following full bleaching regenerates 91% of rhodopsin. The energy densities and the corresponding bleach levels of flashes used in human ORG experiments are summarized in Table S4.

Table S4. Stimulus parameters in human rod ORG experiments.

**Energy density Q_{retina} (photons μm^{-2})**	**Bleach level (%)**
2.83×10^5	0.6
1.70×10^6	3.3
7.79×10^6	14.0
1.65×10^7	27.1
2.65×10^7	39.4

In 1955, Hagins reported that a brief flash (< 1 ms), no matter how strong it is, cannot bleach more than 50% of rhodopsin in the living rabbit eye[31]. This observation was later explained by the ability of early photoproducts, up to Meta I, to absorb a second photon and revert back (photoreversal) to rhodopsin or its isoform (isorhodopsin)[32,33]. Given the millisecond-scale thermal decay time constant of Meta I[34], this effect is expected to be marginal in our current experiments using 5-ms flashes. Nonetheless, a future study that calculates the bleach level considering the entire rhodopsin cycle would be valuable.

5.3 Rhodopsin bleaching caused by the OCT beam

The extent of rhodopsin bleaching induced by the OCT beam in rats and human subjects can be estimated following the approaches described in Supplementary Sections 5.1 and 5.2, respectively.

- In rats, the OCT beam power at the cornea was measured to be 2.0 mW. Considering an illumination area of 800 μm $\times$ 7.2 μm on the retina, the rhodopsin absorption spectrum, and the power spectrum of the OCT light source, Eq. S10 yields an effective energy density Q of 1.70×10^4 photons μm^{-2} s^{-1}, corresponding to a rhodopsin bleach rate of 0.02% per second.
- In human subjects, the OCT beam power at the cornea was measured to be 1.5 mW and distributed over an area of ~0.5 mm^2 on the retina. According to Eq. S12, the effective photon density was calculated to be ~50 photons μm^{-2} s^{-1}. The resulting rhodopsin bleaching (~10^{-6} per second) is substantially lower than that induced by visual stimulation (≥0.6% in 5 ms).

5.4 Radiant exposure safety assessment of flash stimuli in human subjects

According to ANSI Z80.36-2021 (Ophthalmics - Light Hazard Protection for Ophthalmic Instruments), the weighted retinal visible and infrared radiation radiant exposure, $H_{\text{VIR-R}}$, should be less than $3{t_f}^{1/4}$ J cm^{-2} for wavelengths between 380 nm and 1350 nm, and for exposure durations in the range $0.625\ \text{ms} \le t_f < 0.25$ s. Accordingly, $H_{\text{VIR-R}}$ should be limited to less than 0.8 J cm^{-2} for the 5-ms, 470-nm flash stimulus used in human ORG experiments.

Quantitatively, $H_{\text{VIR-R}}$ can be calculated by,

$$H_{\text{VIR-R}} = t_f \cdot \int E(\lambda)R(\lambda)d\lambda \tag{S15}$$

where $E(\lambda)$ is the spectral irradiance, and $R(\lambda)$ is the visible and infrared radiation thermal hazard weighting function, which equals to 2.67 at $\lambda = 470$ nm.

In the human ORG experiments, the maximum visual stimulation power delivered to the eye was 3.74 mW, covering an area of 6.36×10^{-3} cm^2 on the retina. This corresponds to an $H_{\text{VIR-R}}$ of less than 1×10^{-2} J cm^{-2}, well below the permissible limit of 0.8 J cm^{-2}.

Supplementary Figures

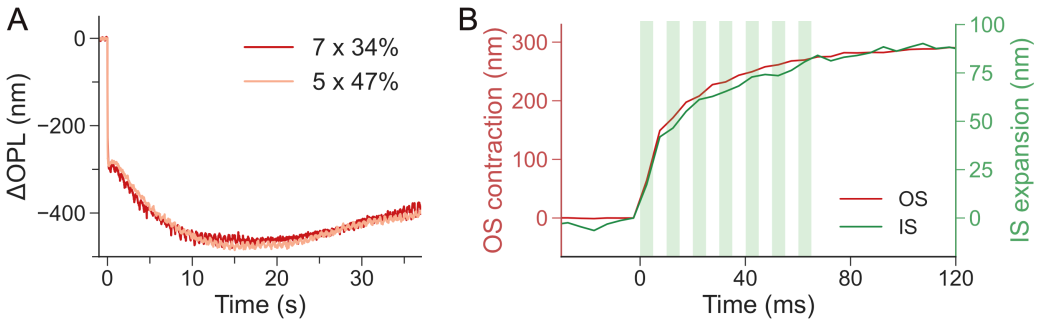

Figure S4. ORG responses in the rod IS and OS evoked by serial flashes with a flash duration of 5 ms and an inter-flash interval of 5 ms. (A) Rod OS responses evoked by two sets of serial-flash stimuli: one with seven flashes, each at a 34% bleach level (red curve), and the other with five flashes, each at a 47% bleach level (pink curve). Both configurations were expected to bleach approximately 95% of the total rhodopsin (94.5% vs 95.8%) by the end of the stimulation process. (B) The rapid OS contraction and the corresponding IS expansion in response to seven flashes, each at a 34% bleach level. The green blocks show the timing of the multiple flash stimuli. The rapid OS responses evoked by each flash in sequence gradually decayed, which followed the expected trend for a smaller number of rhodopsins bleached after each flash. The IS expansion and OS contraction exhibit similar trends.

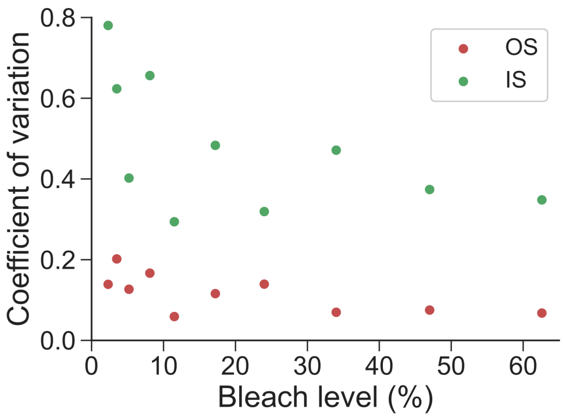

Figure S5. The coefficient of variation (the ratio of the standard deviation to the mean) of the rapid OS contraction amplitude is consistently smaller than that of the rapid IS expansion amplitude.

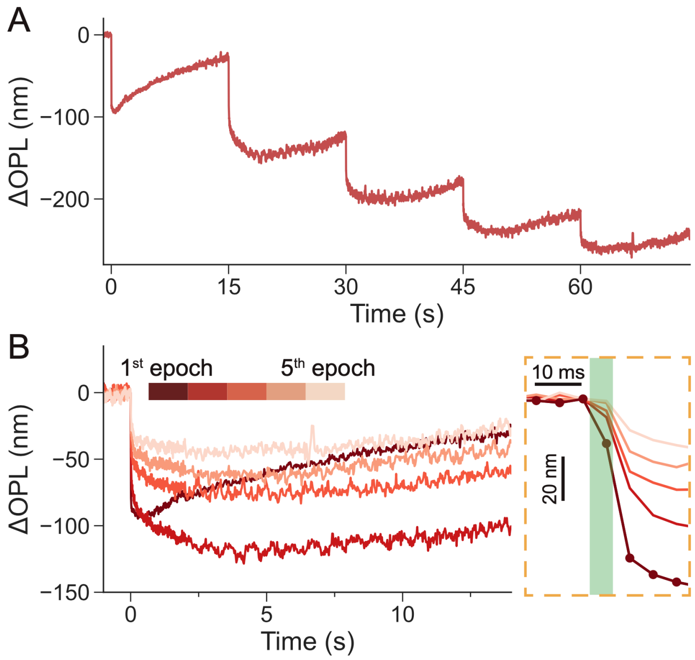

Figure S6. ORG responses in the rod OS elicited by multiple flashes with an inter-flash interval of 15 s, where each flash bleached 8.1% of the remaining rhodopsins. (A) Prolonged ORG recording of the light-evoked rod OS response during the multi-flash stimulation protocol. (B) The rod OS responses extracted from each epoch were aligned to the flash onsets, with the enlarged view shown in the dashed rectangle.

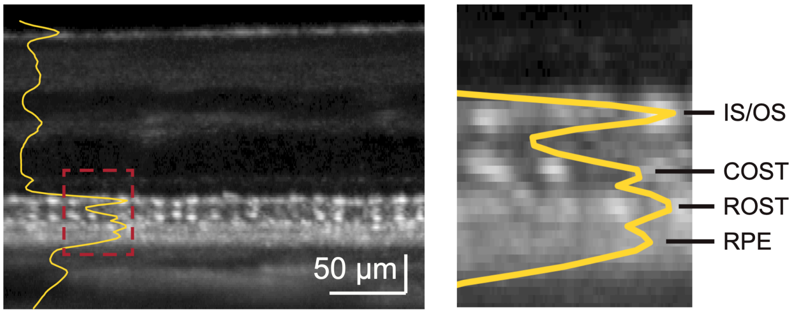

Figure S7. A representative cross-sectional image of a human retina at 10° temporal eccentricity. The yellow curve represents the averaged axial intensity profile. The enlarged view reveals four peaks corresponding to the inner segment/outer segment junction (IS/OS), cone outer segment tip (COST), rod outer segment tip (ROST), and retinal pigment epithelium (RPE)[35,36]. En-face images of ROST (Fig. 5A) and COST (Fig. 5G) were generated by taking the maximum intensity projections of ~10 µm bands centered at their respective reflectance peaks.

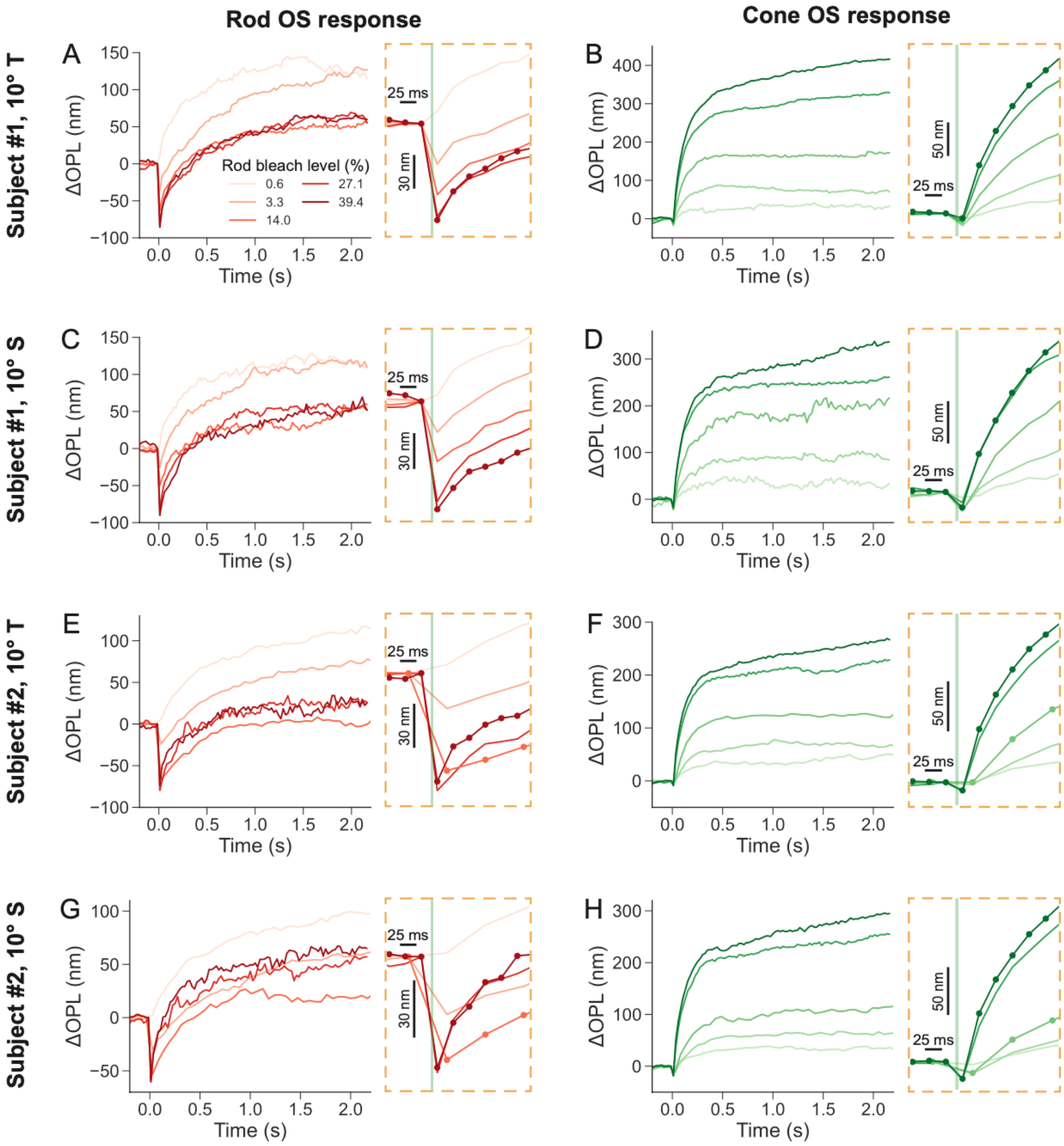

Figure S8. ORG signals in human rod OS and cone OS. Stimulus strengths ranged from $2.8 \times 10^5 - 2.7 \times 10^7$ photons μm^{-2}, corresponding to 0.6% (light color) to 39.4% (deep color) rhodopsin bleach (panel A). Protocol 1, with a volumetric scan rate of 41.4 Hz, was used for the first subject at all bleach levels and for the second subject at 25.0% and 33.9% bleach levels. Protocol 2, with a volumetric scan rate of 17.3 Hz, was used for the second subject at 0.7%, 3.2%, and 12.8% bleach levels.

Supplementary References

1. Tan B*, et al.* Light-evoked deformations in rod photoreceptors, pigment epithelium and subretinal space revealed by prolonged and multilayered optoretinography. *Nat. Commun.* **15**, 5156 (2024).

2. Ou Z*, et al.* Achieving optical transparency in live animals with absorbing molecules. *Science* **385**, eadm6869 (2024).

3. Jagger WS, Liebman PA. Anomalous dispersion of rhodopsin in rod outer segments of the frog. *J. Opt. Soc. Am.* **66**, 56-59 (1976).

4. Kaplan MW. Modeling the rod outer segment birefringence change correlated with metarhodopsin ii formation. *Biophys. J.* **38**, 237-241 (1982).

5. Liebman PA, Jagger WS, Kaplan MW, Bargoot FG. Membrane structure changes in rod outer segments associated with rhodopsin bleaching. *Nature* **251**, 31-36 (1974).

6. de Grip WJ, Ganapathy S. Rhodopsins: An excitingly versatile protein species for research, development and creative engineering. *Frontiers in Chemistry* **10**, (2022).

7. Zhang P, Goswami M, Zawadzki RJ, Pugh EN, Jr. The photosensitivity of rhodopsin bleaching and light-induced increases of fundus reflectance in mice measured in vivo with scanning laser ophthalmoscopy. *Invest. Ophthalmol. Vis. Sci.* **57**, 3650-3664 (2016).

8. Guziewicz KE*, et al.* Best1 gene therapy corrects a diffuse retina-wide microdetachment modulated by light exposure. *Proc. Natl. Acad. Sci. U.S.A.* **115**, E2839 (2018).

9. Lindell M*, et al.* Volumetric reconstruction of a human retinal pigment epithelial cell reveals specialized membranes and polarized distribution of organelles. *Invest. Ophthalmol. Vis. Sci.* **64**, 35-35 (2023).

10. Zhang P*, et al.* In vivo optophysiology reveals that g-protein activation triggers osmotic swelling and increased light scattering of rod photoreceptors. *Proc. Natl. Acad. Sci. U.S.A.* **114**, E2937-E2946 (2017).

11. Pöge M, Mahamid J, Imanishi SS, Plitzko JM, Palczewski K, Baumeister W. Determinants shaping the nanoscale architecture of the mouse rod outer segment. *eLife* **10**, e72817 (2021).

12. Preston GM, Carroll TP, Guggino WB, Agre P. Appearance of water channels in xenopus oocytes expressing red cell chip28 protein. *Science* **256**, 385-387 (1992).

13. Russell ES, Bernstein SE. Blood and blood formation. In: *Biology of the laboratory mouse* (ed Green EL). 2nd edn. McGraw-Hill (1966).

14. Hagins WA, Penn RD, Yoshikami S. Dark current and photocurrent in retinal rods. *Biophys. J.* **10**, 380-412 (1970).

15. Volland S*, et al.* Three-dimensional organization of nascent rod outer segment disk membranes. *Proc. Natl. Acad. Sci. U.S.A.* **112**, 14870-14875 (2015).

16. Burgoyne T, Meschede IP, Burden JJ, Bailly M, Seabra MC, Futter CE. Rod disc renewal occurs by evagination of the ciliary plasma membrane that makes cadherin-based contacts with the inner segment. *Proc. Natl. Acad. Sci. U.S.A.* **112**, 15922-15927 (2015).

17. Mustafi D, Engel AH, Palczewski K. Structure of cone photoreceptors. *Prog. Retin. Eye Res.* **28**, 289-302 (2009).

18. Boyle KC*, et al.* Mechanisms of light-induced deformations in photoreceptors. *Biophys. J.* **119**, 1481-1488 (2020).

19. Lyubarsky AL, Daniele LL, Pugh EN. From candelas to photoisomerizations in the mouse eye by rhodopsin bleaching in situ and the light-rearing dependence of the major components of the mouse erg. *Vision Res.* **44**, 3235-3251 (2004).

20. Makino CL, Taylor WR, Baylor DA. Rapid charge movements and photosensitivity of visual pigments in salamander rods and cones. *J. Physiol. (Lond.)* **442**, 761-780 (1991).

21. Rüppel H, Hagins WA. Spatial origin of the fast photovoltage in retinal rods. In: *Biochemistry and Physiology of Visual Pigments* (ed Langer H). Springer Berlin Heidelberg (1973).

22. Ling T*, et al.* Full-field interferometric imaging of propagating action potentials. *Light Sci. Appl.* **7**, 107 (2018).

23. Hochstrate P, Lindau M, Rüppel H. On the origin and the signal-shaping mechanism of the fast photosignal in the vertebrate retina. *Biophys. J.* **38**, 53-61 (1982).

24. Kessler C, Tillman M, Burns ME, Pugh Jr EN. Rhodopsin in the rod surface membrane regenerates more rapidly than bulk rhodopsin in the disc membranes in vivo. *J. Physiol. (Lond.)* **592**, 2785-2797 (2014).

25. Perlman I. Kinetics of bleaching and regeneration of rhodopsin in abnormal (rcs) and normal albino rats in vivo. *J. Physiol. (Lond.)* **278**, 141-159 (1978).

26. Lamb TD. Photoreceptor spectral sensitivities: Common shape in the long-wavelength region. *Vision Res.* **35**, 3083-3091 (1995).

27. Morgan JIW, Pugh EN, Jr. Scanning laser ophthalmoscope measurement of local fundus reflectance and autofluorescence changes arising from rhodopsin bleaching and regeneration. *Invest. Ophthalmol. Vis. Sci.* **54**, 2048-2059 (2013).

28. Pandiyan VP, Nguyen PT, Pugh EN, Sabesan R. Human cone elongation responses can be explained by photoactivated cone opsin and membrane swelling and osmotic response to phosphate produced by rgs9-catalyzed gtpase. *Proc. Natl. Acad. Sci. U.S.A.* **119**, e2202485119 (2022).

29. Norren DV, Vos JJ. Spectral transmission of the human ocular media. *Vision Res.* **14**, 1237-1244 (1974).

30. Mahroo OAR, Lamb TD. Recovery of the human photopic electroretinogram after bleaching exposures: Estimation of pigment regeneration kinetics. *J. Physiol. (Lond.)* **554**, 417-437 (2004).

31. WA H. The quantum efficiency of bleaching of rhodopsin in situ. *J. Physiol. (Lond.)* **129**, 22-23P (1955).

32. Dowling JE, Hubbard R. Effect of instantaneous flashes on adaptation of the eye: Effects of brilliant flashes on light and dark adaptation. *Nature* **199**, 972-975 (1963).

33. Williams TP. Photoreversal of rhodopsin bleaching. *J. Gen. Physiol.* **47**, 679-689 (1964).

34. Hofmann KP, Lamb TD. Rhodopsin, light-sensor of vision. *Prog. Retin. Eye Res.* **93**, 101116 (2023).

35. Felberer F*, et al.* Adaptive optics slo/oct for 3d imaging of human photoreceptors in vivo. *Biomed. Opt. Express* **5**, 439-456 (2014).

36. Liu Z, Kocaoglu OP, Miller DT. 3d imaging of retinal pigment epithelial cells in the living human retina. *Invest. Ophthalmol. Vis. Sci.* **57**, OCT533-OCT543 (2016).
